# Impact of Premature Birth and Delayed Cuddling on Maternal Support Needs and Satisfaction With Postnatal Care and Changes in Support Over Time

**DOI:** 10.1111/cch.70170

**Published:** 2025-11-20

**Authors:** Achim Fieß, Alica Hartmann, Alexander K. Schuster, Stephanie D. Grabitz, Dirk Wackernagel, Michael S. Urschitz, Jonas Tesarz, Manfred E. Beutel, Mareike Ernst, Eva Mildenberger, Sandra Gißler

**Affiliations:** ^1^ Department of Ophthalmology University Medical Centre of the Johannes Gutenberg University Mainz Mainz Germany; ^2^ Division of Neonatology, Department of Paediatrics University Medical Centre of the Johannes Gutenberg University Mainz Mainz Germany; ^3^ Division of Paediatric Epidemiology, Institute of Medical Biostatistics, Epidemiology, and Informatics University Medical Centre of the Johannes Gutenberg University Mainz Mainz Germany; ^4^ Department of Psychosomatic Medicine and Psychotherapy University Medical Centre of the Johannes Gutenberg University Mainz, University Medical Center Mainz Mainz Germany; ^5^ DZPG (German Centre for Mental Health—Partner Site Heidelberg/Mannheim/Ulm) Heidelberg Germany; ^6^ Department of Clinical Psychology, Psychotherapy and Psychoanalysis, Institute of Psychology University of Klagenfurt Klagenfurt am Wörthersee Austria

**Keywords:** delayed cuddling, maternal and paternal perceptions, parental support needs, postnatal care quality, preterm birth

## Abstract

**Background:**

Preterm birth and early bonding disruptions such as delayed first cuddling may increase parental vulnerability and support needs in the postnatal period. However, little is known about how these factors interact to shape paternal perceptions of care and unmet support needs across different domains.

**Methods:**

This retrospective cohort study drew on data from 1559 individuals aged 4–52 years and linked parental reports from 940 mothers and 614 fathers. Participants were categorised by gestational age of the children into extremely preterm (≤ 28 weeks), very preterm (29–32 weeks), moderately preterm (33–36 weeks) and term (≥ 37 weeks). Multivariable logistic regression was used to examine associations between gestational age, birth weight percentile and delayed first cuddling with maternal desires for administrative, social and medical support. Perceived quality of care from healthcare providers was also assessed.

**Results:**

Mothers of preterm infants who experienced delayed cuddling reported significantly higher needs for administrative, social and medical support compared to mothers of term infants. Delayed cuddling emerged as a consistent predictor of increased support needs across all domains. Mothers of preterm infants were more likely to rate physician care positively, while delayed cuddling and low birth weight percentile were associated with lower satisfaction with midwifery care. Fathers showed similar but less pronounced patterns, with administrative support needs elevated in preterm groups.

**Conclusion:**

Preterm birth and delayed first cuddling are associated with greater maternal support needs and lower satisfaction with certain aspects of care, particularly midwifery services. These findings underscore the importance of early, well‐coordinated and responsive postnatal support structures, especially for mothers of preterm infants and those who experienced bonding disruptions. Tailored interventions addressing administrative, social and emotional support needs may help reduce long‐term stress and improve parent–infant outcomes.

## Summary

- Mothers of preterm infants and those who experienced delayed first cuddling report greater unmet needs for administrative, social and medical support.
- Satisfaction with postnatal care varies by provider type, with lower ratings for midwifery care linked to delayed cuddling.
- Paternal perceptions of support show similar but less pronounced patterns, underlining the need to include fathers more systematically in research and support planning.
- Tailored postnatal interventions should account for early birth and first physical contact experiences and address both maternal and paternal support needs to improve family outcomes.

## 1 Introduction

Prematurity is a global problem affecting around 13.4 million newborns in 2020 (Ohuma et al. 2023). These children often require more intensive medical care, which is associated with longer hospital stays (Maier et al. 2018) and long‐term health problems (Markopoulou et al. 2019). Therefore, their parents may face particular challenges, possibly accompanied by a high level of emotional stress (Rafael‐Gutiérrez et al. 2020; Ionio et al. 2016) and the risk of mental health problems (Pace et al. 2016). Consequently, individuals born preterm and their parents need medical and psychosocial support to counteract negative sequelae that shape development and unfavourable outcomes over their life span.

A growing body of evidence has highlighted postnatal care, particularly in supporting parent–child bonding, as an important protective factor. Early skin‐to‐skin contact, commonly known as *Kangaroo Care*, has been proven to mitigate the health risks associated with premature birth while also alleviating the emotional strain on parents. This practice supports newborn health by stabilising their heart rate (Swieter et al. 2023), enhancing respiratory function (Lee et al. 2022) and promoting healthy weight gain (Shattnawi and Al‐Ali 2019). For parents, it helps to reduce the anxiety that often accompanies the unexpected challenges of premature birth, fostering a sense of empowerment and strengthening the long‐term bond with their child (Cong et al. 2021; Lilliesköld et al. 2022). Nonetheless, many parents of premature babies report that current support programmes do not adequately address their needs, particularly in facilitating Kangaroo Care (Foong et al. 2023).

Current support programmes for vulnerable groups of parents, such as parents of premature babies, are not sufficiently developed. A study of 27 parents of extremely premature babies (gestational age 23–27 weeks) found that psychosocial needs were sometimes unmet due to staff shortages or insufficient communication. Parents reported weakened trust in healthcare staff and a desire for structured emotional support (Bry and Wigert 2019). Financial issues after discharge from the hospital and the neonatal intensive care unit are also a burden for parents. A survey of 365 parents of premature babies (gestational age: < 37 weeks) showed that 53% of participants sometimes or often worried about health costs. Unexpected costs such as the use of medical equipment contributed to this, as did the loss of time at work due to the increased care needs of premature babies. It is of note that there are differences in the financial burden depending on the geographical location (Lakshmanan et al. 2022).

Therefore, this study was designed to investigate the support needs and experiences of mothers and fathers of premature infants. Particular attention was given to initial cuddling with the child and to parents' wishes for administrative, medical, social and economic support. How these support needs were influenced by factors such as the degree of prematurity and fetal growth restriction was also explored. In addition, we examined how well the care provided by different healthcare professionals was perceived in order to identify potential areas for improvement. This study provides a more nuanced analysis by differentiating between various degrees of prematurity, birth weight percentiles and experiences of delayed cuddling to identify more precise and effective ways to support these vulnerable families.

## 2 Methods

### 2.1 Study Population

The Gutenberg Prematurity Study (GPS) and the Gutenberg Prematurity Study Young (GPSY) are retrospective cohort studies with prospective examinations and parental interviews conducted. The original cohorts consisted of individuals born preterm or at term between 1969 and 2018, who were aged 4–52 years at the time of enrolment (see Figure S1). For the present study, the focus was on their mothers and fathers, who were invited to participate in interviews regarding their experiences and support needs following the preterm or term birth of their child.

The original index cohort recruitment was as follows: invitations were sent to every second individual born preterm from 1969 onward with a gestational age of 33–36 weeks and to all individuals born preterm at 32 weeks of gestation or less at the University Medical Center Mainz (UMCM). For each birth month from 1969 to 2018, we selected six individuals born at term, three male and three female, with a birth weight between the 10th and 90th percentile for the GPS cohort. In addition, we selected eight full‐term infants, four male and four female, for the GPSY cohort (Fieß, Gißler, et al. 2024, Fieß et al. 2022, Fieß, Hartmann, et al. 2024, Fieß et al. 2023). To facilitate stratification of the effects of varying levels of abnormal perinatal fetal nutrition independent of prematurity, 70 full‐term participants were invited from each of the following categories: severely small for gestational age (SGA; birth weight percentile below the 3rd percentile), moderately SGA (birth weight percentile between the 3rd and 10th), moderately large for gestational age (LGA; birth weight percentile between the 90th and 97th) and severely LGA (birth weight percentile above the 97th). These individuals served as the basis for identifying and inviting their parents to participate, ensuring a similar distribution of age and gender across groups.

The primary focus of the present study was on the parents of individuals. Parents were grouped according to their child's gestational age at birth, so the parental participants were grouped as follows: Group 1 included parents with children born at 37 + 0 weeks or later, Group 2 consisted of parents with children born from 33 + 0 to 36 + 6 weeks, Group 3 contained parents with children born from 29 + 0 to 32 + 6 weeks and Group 4 assessed parents with children born at or before 28 + 6 weeks. In total, 418 mothers participated in Group 1, 230 in Group 2, 171 in Group 3 and 121 in Group 4. There were 276 fathers in Group 1, 161 in Group 2, 96 in Group 3 and 81 in Group 4. Due to the lower number of fathers, the main analysis focused on mothers. The majority of participants were of White ethnicity.

All participants provided written informed consent prior to joining the study, adhering to the standards of Good Clinical Practice, Good Epidemiological Practice and the ethical guidelines of the Declaration of Helsinki. All children and parents gave permission to link their data. The study's protocol and related documents received approval from the local ethics committee of the Medical Chamber of Rhineland‐Palatinate, Germany, under Reference No. 2019‐14161 (original approval: 29.05.2019; most recent update: 02.04.2020) and Reference No. 2021‐15830 (original approval: 05.05.2021; latest update: 19.01.2022).

### 2.2 Data Collection

As part of the ongoing GPS and GPSY cohort studies, children, adolescents and adults were initially assessed using standardised instruments. For underage participants, their parents were invited to participate directly as part of the study procedures. In the case of adult participants, parental contact was only initiated if the adult child consented and provided their parents' contact information. Recruitment and data collection took place between 2019 and 2023 under the coordination of the UMCM.

Participants were given the option to complete the sociodemographic and clinical questionnaire either online via LimeSurvey or as a paper‐based version returned by mail. The majority of questionnaires were completed at home. For participants who required support or preferred in‐person assistance, the questionnaire was completed on site during their scheduled visit. Study staff were available to answer any questions and provide clarification where needed. The questionnaire included both standardised instruments and custom‐designed items. Parental support needs were assessed through structured questions developed by the study team, asking, for example, whether mothers would have wished for more administrative, medical, social or economic support after birth and from whom (e.g., family, social workers, nurses and paediatricians). These items were derived from clinical experience and expert consensus but were not based on previously validated scales. In contrast, the German version of the Paediatric Integrated Care Survey (PICS‐D) is a validated tool and was administered as part of the written questionnaire to a subgroup of participants to assess satisfaction with aspects of integrated care. Participants completed the instrument in its original German version.

Following questionnaire completion, participants underwent a structured clinical interview conducted by trained staff to verify and supplement the information provided. Further data such as time to first physical contact (cuddling) were cross‐checked with maternal medical records when available. All collected data were pseudonymised at entry and stored on secure servers with restricted access. Personally identifying information was kept separate and managed in line with institutional and national data protection regulations to ensure participant confidentiality.

Parents were asked various questions about their experiences of support after birth. They could tick a box to indicate whether they desired more administrative, medical, social or economic support (e.g., ‘What kind of additional support or care would you have wished for regarding your newborn child?’ with response options such as administrative, medical, social or economic). Similarly, they were asked to indicate from whom they would have wished for more support (e.g., ‘From whom or which services would you have wished for more support?’, including family members, social workers, nursing services or paediatricians). In addition, parents were asked open‐ended questions about the quality of care after hospital discharge, with separate prompts regarding physicians, nurses and midwives (e.g., ‘What did you find good or bad about the care your child received?’). For the purpose of statistical analysis, responses were recoded into binary variables indicating whether participants positively evaluated the care provided by each professional group.

The PICS‐D, a validated questionnaire assessing paediatric care integration experiences, was used in this study. It evaluates various aspects of integrated care, including the coordination of medical and social support services and the satisfaction of parents with the care provided. The survey covers key areas such as the quality of interactions with healthcare professionals, the adequacy of support from family and healthcare providers and overall parental satisfaction with the care their child received. The questionnaire comprises three main facets: Team Quality & Communication, Impact on the Family and Access to Care but Access to Care was not used due to insufficient psychometric properties in previous investigations (Willems et al. 2022). To explore trends over time in perceived maternal support, a composite score was computed by summing all items from the two validated subscales ‘Team Quality & Communication’ and ‘Impact on the Family’ of the PICS‐D. This aggregated indicator served solely for descriptive visualisation of the longitudinal trend and was not used in any inferential analyses. No modifications were made to the original PICS‐D items. Each item was scored using a Likert scale ranging from 1 to 6 (1 = *never*, 6 = *always*) with higher scores indicating greater satisfaction with the quality of care. A full list of the items is provided in Table S1.

### 2.3 Evaluation of Prenatal and Postnatal History

The medical records of the offspring, archived at the UMCM, were reviewed to extract information including gestational age (in weeks) and birth weight (in kilograms). Additional factors were assessed through medical chart reviews and maternal interviews including placental insufficiency, maternal smoking, preeclampsia and breastfeeding history. Birth weight percentiles were calculated according to Voigt et al. (2006).

### 2.4 Statistical Analysis

The absolute and relative frequencies were determined for dichotomous parameters, with the mean and standard deviation calculated for normally distributed variables and the median and interquartile range for non‐normally distributed variables. Differences across gestational age groups were evaluated by one‐way ANOVA for continuous variables and the chi‐square test for categorical variables, calculating global *p* values.

Multivariable logistic regression was applied to analyse primary outcomes reported by the mothers: more administrative support (yes), more desired support medically (yes), more social support (yes) or economically (yes) and good satisfaction with the care of physicians (yes), nurses (yes) and midwifery care (yes). The independent variables were analysed as follows: Model 1 included gestational age categories (≤ 28, 29–32 and 33–36 weeks); Model 2 included gestational age categories and birth weight percentile; and Model 3 included gestational age categories, birth weight percentile and weeks until first cuddling. A gestational age of ≥ 37 weeks was used as the reference category for the gestational age groups. Gestational age was categorised into clinically meaningful groups following the WHO classification for preterm birth (WHO 2018) with a slight modification to the definition of extremely preterm birth to ensure a sufficient number of cases in this category. This stratification allows for a differentiated analysis of parental support needs in relation to the severity of prematurity and associated clinical complexity. Birth weight percentile was included as a continuous variable in all multivariable analyses to capture nuanced variations in fetal growth across the full spectrum. This approach allows for a more sensitive assessment of how subtle differences in birth weight percentile relate to parental support experiences, independent of gestational age. Weeks until first cuddling was included as a continuous variable to capture early bonding opportunities, which may influence maternal satisfaction and perceived support. All analyses were adjusted for parental age and the age of the child. This adjustment was implemented to account for potential recall bias, as well as for temporal changes in healthcare services that may have influenced maternal perceptions and experiences. By statistically controlling for these variables, we aimed to reduce confounding effects related to differences in the time elapsed since birth and shifts in clinical practices over the past decades. Only those participants with both maternal and paternal questionnaires completed were included for a direct comparison of associations between parents, examining the differences in perceptions and outcomes between mothers and fathers. Due to the smaller number of paternal data, weeks of prematurity (40 minus gestational age) were used as a continuous variable instead of categorical gestational age groups to ensure sufficient statistical power (Table S2).

Finally, a subgroup analysis was conducted on the mothers of children aged 4–17 years using the PICS‐D questionnaire (*n* = 395) with a total score calculated to reflect the overall level of perceived support. This score indicates the degree of support that mothers felt they received during the postnatal period. Focusing on this subgroup allowed for a more homogeneous sample in terms of time since birth and reduced the risk of recall bias, thereby ensuring a more robust and interpretable analysis of perceived support, which was explored descriptively through graphical data visualisation.

This is an exploratory study, so no adjustment for multiple testing was performed. All statistical analyses were conducted using R Version 4.3.2.

## 3 Results

### 3.1 Descriptive Characteristics and Parental Support Experiences by Gestational Age

Perinatal and demographic characteristics of the 1559 offspring (median age 15 years, range: 4–52 years; 824 females) are presented in Table 1. Across gestational age groups, the proportions reporting the need for administrative, social and medical support differed significantly, with higher proportions in the preterm groups (Table 2). These groups also voiced a greater desire for support from social workers and paediatricians. Mothers of preterm infants reported more frequently taking up professional help compared to those of full‐term infants. The groups also differed regarding satisfaction with care, with the largest proportion among those with very preterm infants being content with the care received by physicians. Satisfaction with midwifery care was most common among those whose babies were born at term.

**TABLE 1:** Perinatal characteristics of index individuals by gestational age group (*N* = 1559).

	Group 1	Group 2	Group 3	Group 4
	GA ≥ 37 weeks	GA 33–36 weeks	GA 29–32 weeks	GA ≤ 28 weeks
Number of children	680	379	296	204
Female	354 (52.1%)	202 (53.3%)	155 (52.4%)	113 (55.4%)
Age (years), median (IQR)	16.0 [11.0, 27.0]	15.0 [10.0, 23.0]	16.0 [11.0, 24.0]	14.0 [9.0, 18.3]
Birth weight (g), mean (SD)	3407.21 ± 764.56	2238.11 ± 448.51	1535.61 ± 364.96	826.67 ± 240.82
BW < 1500 g	2 (0.3%)	20 (5.3%)	136 (45.9%)	204 (100%)
BW < 1000 g	0 (0.0%)	0 (0.0%)	23 (7.8%)	154 (75.5%)
BW percentile, mean (SD)	47.68 ± 35.03	30.83 ± 23.66	41.17 ± 24.31	37.62 ± 26.05
Gestational age (weeks), mean (SD)	39.09 ± 1.4	34.52 ± 1.0	30.70 ± 1.1	25.89 ± 1.6

Abbreviations: IQR—interquartile range; SD—standard deviation.

**TABLE 2:** Mothers' experiences of support after their child's birth.

	Group 1	Group 2	Group 3	Group 4	*p* value^a^
	GA ≥ 37 weeks	GA 33–36 weeks	GA 29–32 weeks	GA ≤ 28 weeks	
Maternal participants	418	230	171	121	
Age at assessment (years)	50.1 ± 9.9	47.9 ± 9.2	49.0 ± 8.6	46.8 ± 9.2	0.003
More support desired (yes)					
Administrative	13 (3.2%)	13 (5.9%)	24 (14.6%)	15 (13.3%)	< 0.001
Social	49 (12.2%)	34 (15.3%)	45 (27.4%)	37 (32.7%)	< 0.001
Medical	42 (10.4%)	46 (20.7%)	39 (23.8%)	27 (23.9%)	< 0.001
Economical	37 (9.2%)	21 (9.5%)	24 (14.6%)	14 (12.4%)	0.23
More support desired by specific groups (yes)					
Family	51 (13.1%)	27 (12.2%)	15 (9.0%)	15 (12.6%)	0.60
Social worker	16 (4.1%)	5 (2.3%)	13 (7.9%)	11 (9.2%)	0.01
Nursing service	22 (5.6%)	18 (8.2%)	16 (9.6%)	8 (6.7%)	0.35
Paediatricians	31 (8.0%)	20 (9.0%)	28 (16.9%)	17 (14.4%)	0.01
Satisfaction with medical care (yes)					
Good care by physicians (yes)	191 (47.4%)	135 (60.8%)	97 (59.1%)	72 (63.7%)	0.001
Good care by nurses (yes)	92 (22.9%)	56 (25.2%)	43 (26.2%)	31 (27.4%)	0.71
Good midwifery care (yes)	76 (18.9%)	30 (13.5%)	30 (18.3%)	8 (7.1%)	0.01
Professional help taken up (yes)	35 (8.6%)	17 (7.7%)	24 (14.3%)	20 (17.2%)	0.01

^a^ One‐way ANOVA was used for continuous variables and the chi‐square test for categorical variables, calculating global *p* values. Percentages are based on the number of valid responses, which vary slightly due to missing data.

The descriptive comparison among fathers also revealed a significantly higher desire for administrative support in the preterm groups (Table 3). In contrast to mothers, there was no difference in the fathers' desire for social and medical support. Similar to mothers, they reported a stronger desire for support from social workers and paediatricians. Furthermore, fathers of preterm infants reported a greater need for support from nursing services, a difference that was not observed among mothers. Fathers of preterm infants reported higher satisfaction with care from physicians and nurses.

**TABLE 3:** Paternal experiences of support after the birth of the child.

	Group 1	Group 2	Group 3	Group 4	*p* value^a^
	GA ≥ 37 weeks	GA 33–36 weeks	GA 29–32 weeks	GA ≤ 28 weeks	
Paternal participants	276	161	96	81	
Age (years)	51.9 ± 10.4	50.7 ± 9.0	50.2 ± 8.4	49.7 ± 9.8	0.21
More support desired (yes)					
Administrative	4 (1.5%)	5 (3.4%)	3 (3.3%)	7 (9.2%)	0.01
Social	16 (6.1%)	14 (9.5%)	7 (7.7%)	10 (13.2%)	0.22
Medical	17 (6.5%)	18 (12.2%)	13 (14.3%)	10 (13.2%)	0.07
Economical	22 (8.4%)	22 (8.4%)	8 (8.8%)	9 (11.8%)	0.75
More support desired in specific groups (yes)					
Family	17 (6.5%)	6 (4.1%)	6 (6.6%)	7 (9.2%)	0.49
Social worker	3 (1.1%)	4 (2.7%)	3 (3.3%)	9 (11.8%)	< 0.001
Nursing service	5 (1.9%)	11 (7.4%)	3 (3.3%)	7 (9.2%)	0.01
Paediatricians	8 (3.1%)	11 (7.4%)	2 (2.2%)	8 (10.5%)	0.02
Satisfaction with medical care					
Good care by physicians (yes)	102 (38.9%)	68 (45.9%)	50 (54.9%)	50 (65.8%)	< 0.001
Good care by nurses (yes)	94 (35.9%)	67 (45.3%)	55 (60.4%)	46 (60.5%)	< 0.001
Good midwifery care (yes)	13 (21.3%)	3 (8.6%)	2 (16.7%)	0 (0.0%)	0.16

^a^ One‐way ANOVA was used for continuous variables and the chi‐square test for categorical variables, calculating global *p* values. Percentages are based on the number of valid responses, which vary slightly due to missing data.

### 3.2 Multivariable Regression Analysis

**FIGURE 1:**
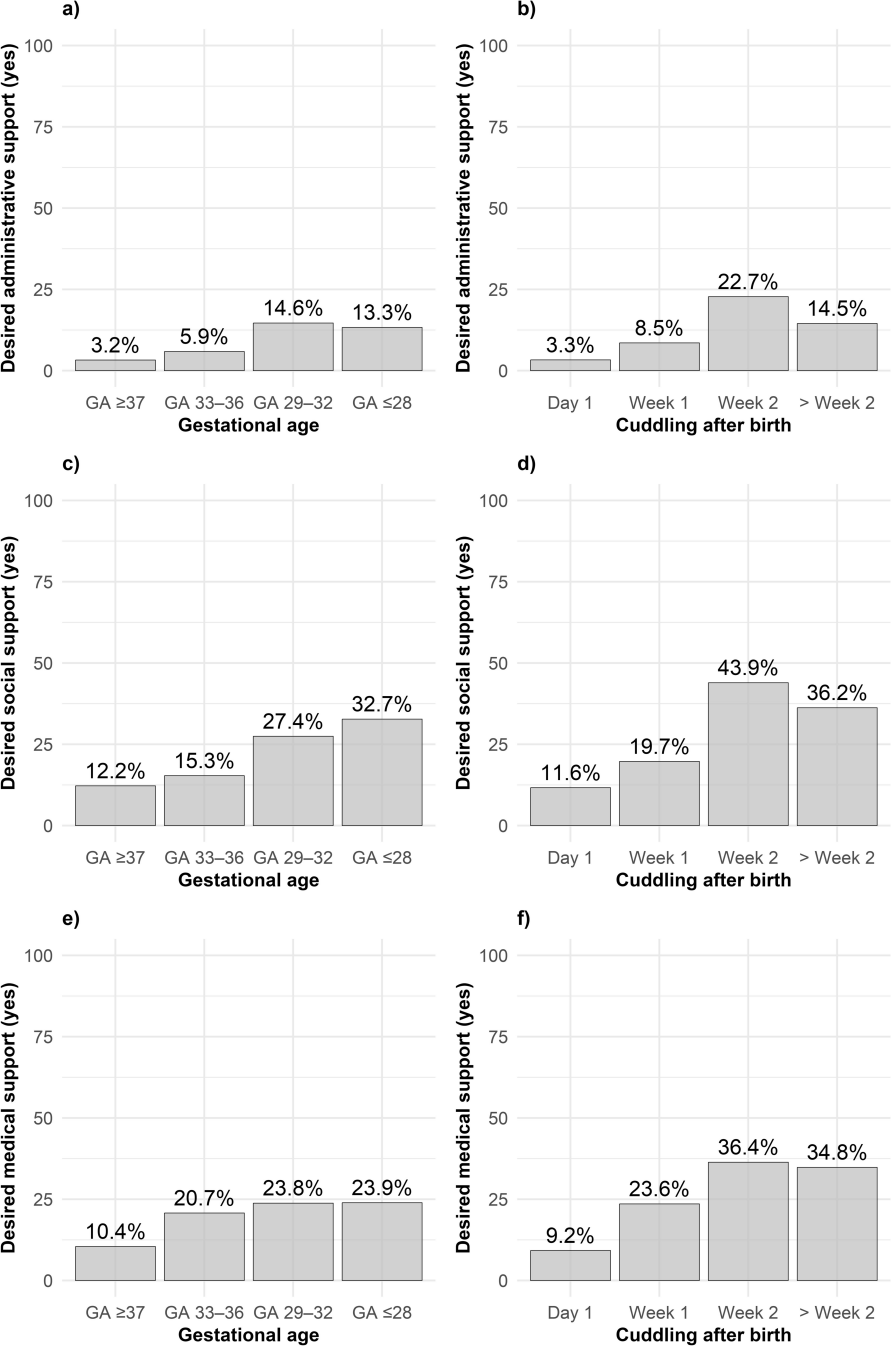
Maternal experience of support stratified by various factors: (a) more administrative support desired (yes) by gestational age groups, (b) by time to first cuddling after birth (weeks), (c) more social support desired by gestational age groups, (d) by time to first cuddling after birth (weeks), (e) more medical support desired by gestational age groups and (e) by time to first cuddling after birth (weeks). *Note:* ‘Week 1’ refers to Days 2–7 after birth, ‘Week 2’ to Days 8–14 and ‘> Week 2’ to Day 15 and later.

The following results refer to multivariable logistic regression analyses based on Model 3, which included gestational age groups, birth weight percentile and delayed cuddling as independent variables. The analysis revealed that mothers with children born at a gestational age of 29–32 weeks (OR = 4.32, 95% CI = 2.05–9.40, *p* < 0.001) and those who reported delayed cuddling (OR = 1.21, 95% CI = 1.03–1.42, *p* = 0.02) had higher odds of reporting a need for more administrative support. For mothers of extremely preterm infants (≤ 28 weeks), this association was elevated but not statistically significant (OR = 2.33, 95% CI = 0.90–5.95, *p* = 0.08) (Table 4 and Figure 1). Regarding social support, mothers of children born at ≤ 28 (OR = 2.28, 95% CI = 1.22–4.22, *p* = 0.01) and 29–32 weeks (OR = 1.93, 95% CI = 1.14–3.26, *p* = 0.01) had higher odds of reporting a need for more social support. Mothers who experienced delayed cuddling also had higher odds of desiring more social support (OR = 1.31, 95% CI = 1.15–1.52, *p* < 0.001). For medical support, mothers of extremely preterm infants (≤ 28 weeks) (OR = 1.99, 95% CI = 1.02–3.80, *p* = 0.04), moderately preterm infants (29–32 weeks) (OR = 2.25, 95% CI = 1.30–3.86, *p* = 0.003) and late preterm infants (33–36 weeks) (OR = 2.13, 95% CI = 1.31–3.48, *p* = 0.002) had higher odds of reporting need than mothers of term infants. A significant association was also observed for delayed cuddling (OR = 1.20, 95% CI = 1.06–1.37, *p* = 0.004).

**TABLE 4:** Association analyses of the experiences of support after the birth of the child in mothers of children born preterm and full‐term adjusted for the mother's and child's age.

	Model 1: Original model		Model 2: Nutritional status		Model 3: Postnatal course	
	OR (95% CI)	*p* value	OR (95% CI)	*p* value	OR (95% CI)	*p* value
More administrative support						
GA ≤ 28 weeks	4.39 (2.01, 9.71)	< 0.001	4.16 (1.89, 9.26)	< 0.001	2.33 (0.90, 5.95)	0.08
GA 29–32 weeks	1.67 (2.68, 11.09)	< 0.001	5.24 (2.63, 10.89)	< 0.001	4.32 (2.05, 9.40)	< 0.001
GA 33–36 weeks	5.34 (0.74, 3.77)	0.21	1.55 (0.68, 3.52)	0.29	1.38 (0.59, 3.16)	0.45
BW percentile			0.99 (0.98, 1.00)	0.30	0.99 (0.98, 1,00)	0.32
Delayed cuddling after birth (weeks)					1.21 (1.03, 1.42)	0.02
More social support						
GA ≤ 28 weeks	3.64 (2.21, 6.00)	< 0.001	3.63 (2.18, 6.01)	< 0.001	2.28 (1.22, 4.22)	0.01
GA 29–32 weeks	2.80 (1.76, 4.46)	< 0.001	2.80 (1.76, 4.45)	< 0.001	1.93 (1.14, 3.26)	0.01
GA 33–36 weeks	1.31 (0.80, 2.11)	0.27	1.30 (0.79, 2.12)	0.29	1.10 (0.65, 1.82)	0.72
BW percentile			1.00 (0.99, 1.01)	0.94	1.00 (0.99, 1.01)	0.96
Delayed cuddling after birth (weeks)					1.31 (1.15, 1.52)	< 0.001
More medical support						
GA ≤ 28 weeks	2.76 (1.59, 4.73)	< 0.001	2.89 (1.66, 5.01)	< 0.001	1.99 (1.02, 3.80)	0.04
GA 29–32 weeks	2.87 (1.77, 4.67)	< 0.001	2.95 (1.81, 4.81)	< 0.001	2.25 (1.30, 3.86)	0.003
GA 33–36 weeks	2.31 (1.46, 3.67)	< 0.001	2.48 (1.54, 4.00)	< 0.001	2.13 (1.31, 3.48)	0.002
BW percentile			1.00 (1.00, 1.01)	0.26	1.00 (1.00, 1.01)	0.38
Delayed cuddling after birth (weeks)					1.20 (1.06, 1.37)	0.004
More economic support						
GA ≤ 28 weeks	1.49 (0.75, 2.84)	0.24	1.55 (0.77, 2.98)	0.20	1.09 (0.46, 2.39)	0.84
GA 29–32 weeks	1.87 (1.06, 3.25)	0.03	1.91 (1.08, 3.32)	0.02	1.70 (0.90, 3.16)	0.09
GA 33–36 weeks	1.11 (0.62, 1.94)	0.73	1.17 (0.64, 2.09)	0.60	1.09 (0.59, 1.98)	0.78
BW percentile			1.00 (1.00, 1.01)	0.43	1.00 (1.00, 1.01)	0.40
Delayed cuddling after birth (weeks)					1.13 (0.99, 1.30)	0.07

*Note:* Model 1 (original) is adjusted for the age (years) of the mother and the age (years) of the child. Model 2 (nutritional status) is additionally adjusted for birth weight percentile. Model 3 (postnatal clinical course) is adjusted for the age (years) of the mother, age (years) of the child, birth weight percentile and cuddling after birth (weeks). A gestational age of ≥ 37 weeks was used as the reference category for the gestational age groups. Models 1–3 were adjusted for maternal age. Adjusted odd ratios (OR) with 95% confidence intervals (95% CI).

Abbreviations: BW—birth weight; GA—gestational age.

**FIGURE 2:**
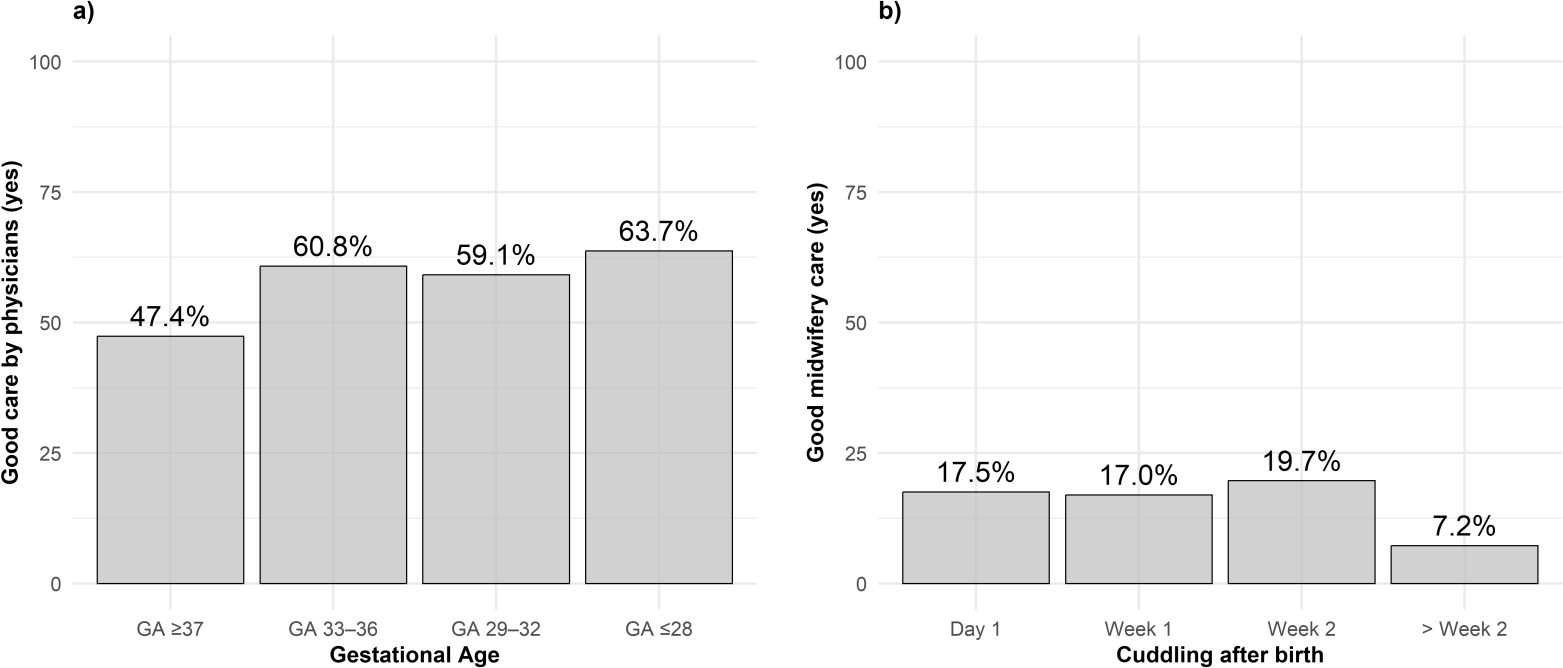
Maternal satisfaction with care: (a) good care by physicians stratified by gestational age and (b) good midwifery care stratified by time to first cuddling after birth. *Note:* ‘Week 1’ refers to Days 2–7 after birth, ‘Week 2’ to Days 8–14 and ‘> Week 2’ to Day 15 and later.

Mothers of preterm birth groups were more likely to report good care provided by physicians compared to mothers of term infants, with higher odds observed in the extremely preterm group (OR = 2.35, 95% CI = 1.37–4.09, *p* = 0.002), the moderately preterm group (OR = 1.81, 95% CI = 1.18–2.81, *p* = 0.01) and the late preterm group (OR = 1.78, 95% CI = 1.24–2.56, *p* = 0.002) (Table 5 and Figure 2). No statistically significant differences between gestational age groups were observed for satisfaction with care provided by nurses. With respect to midwifery care, mothers who experienced delayed cuddling were less likely to report good care (OR = 0.79, 95% CI = 0.62–0.96, *p* = 0.03), while higher birth weight percentile was associated with greater satisfaction with midwifery care (OR = 1.01, 95% CI = 1.00–1.01, *p* = 0.04).

**TABLE 5:** Association analyses of satisfaction with support after the child's birth in mothers of children born preterm and full‐term adjusted for the mother's and child's age.

	Model 1: Original model		Model 2: Nutritional status		Model 3: Postnatal course	
	OR (95% CI)	*p* value	OR (95% CI)	*p* value	OR (95% CI)	*p* value
Good care by physicians						
GA ≤ 28 weeks	2.04 (1.33, 3.18)	0.001	1.98 (1.28, 3.09)	0.002	2.35 (1.37, 4.09)	0.002
GA 29–32 weeks	1.67 (1.15, 2.44)	0.01	1.65 (1.13, 2.41)	0.01	1.81 (1.18, 2.81)	0.01
GA 33–36 weeks	1.79 (1.28, 2.51)	< 0.001	1.71 (1.21, 2.42)	0.002	1.78 (1.24, 2.56)	0.002
BW percentile			1.00 (0.99, 1.00)	0.22	1.00 (0.99, 1.00)	0.17
Delayed cuddling after birth (weeks)					1.01 (0.90, 1.14)	0.90
Good care by nurses						
GA ≤ 28 weeks	1.64 (0.99, 2.69)	0.05	1.57 (0.94, 2.57)	0.08	1.36 (0.73, 2.49)	0.32
GA 29–32 weeks	1.30 (0.83, 2.03)	0.25	1.27 (0.81, 1.98)	0.29	1.22 (0.73, 2.03)	0.43
GA 33–36 weeks	1.36 (0.91, 2.04)	0.13	1.26 (0.83, 1.91)	0.28	1.19 (0.77, 1.85)	0.43
BW percentile			1.00 (0.99, 1.00)	0.11	1.00 (0.99, 1.00)	0.16
Delayed cuddling after birth (weeks)					1.06 (0.94, 1.20)	0.37
Good midwifery care						
GA ≤ 28 weeks	0.35 (0.15, 0.72)	0.008	0.38 (0.16, 0.78)	0.01	0.71 (0.28, 1.61)	0.43
GA 29–32 weeks	1.05 (0.64, 1.68)	0.84	1.10 (0.67, 1.76)	0.71	1.58 (0.89, 2.77)	0.11
GA 33–36 weeks	0.72 (0.34, 1.14)	0.16	0.81 (0.50, 1.31)	0.40	1.03 (0.61, 1.71)	0.91
BW percentile			1.01 (1.00, 1.01)	0.03	1.01 (1.00, 1.01)	0.04
Delayed cuddling after birth (weeks)					0.79 (0.62, 0.96)	0.03

*Note:* Model 1 (original) is adjusted for the age (years) of the mother and the age (years) of the child. Model 2 (nutritional status) is additionally adjusted for birth weight percentile. Model 3 (postnatal clinical course) is adjusted for the age (years) of the mother, age (years) of the child, birth weight percentile and cuddling after birth (weeks). A gestational age of ≥ 37 weeks was used as the reference category for the gestational age groups. Models 1–3 were adjusted for maternal age and the age of the child. Adjusted odd ratios (OR) with 95% confidence intervals (95% CI).

Abbreviations: BW—birth weight; GA—gestational age.

In fathers, lower gestational age showed a similar tendency towards higher administrative support need (OR = 1.12, 95% CI = 1.00–1.26, *p* = 0.06), though this association did not reach statistical significance (Table S2). Fathers did not report a significantly increased desire for social support in relation to gestational age (OR = 1.03, 95% CI = 0.95–1.13, *p* = 0.45). Fathers who experienced delayed cuddling had higher odds of reporting social support need (OR = 1.29, 95% CI = 1.00–1.65, p = 0.04). For medical support, later cuddling was associated with higher odds among mothers (OR = 1.37, 95% CI = 1.12–1.71, *p* = 0.003) but not among fathers (OR = 1.06, 95% CI = 0.80–1.34, *p* = 0.64).

In a descriptive subgroup analysis of mothers of children aged 4–17 years, we visually explored the relationship between the child's birth year and the mother's perceived support from the care network, as measured by the total PICS‐D score. Figure S2 illustrates a declining trend, showing that mothers of more recently born children tended to report lower levels of support compared to those whose children were born earlier.

## 4 Discussion

This study examined associations between premature birth, delayed cuddling and maternal perceptions of postnatal support. Mothers of extremely to moderately preterm infants and those who experienced delayed cuddling reported a greater need for administrative, social and medical support compared to mothers of term‐born infants or those with shorter times to first cuddle. Mothers of extremely to moderately preterm infants reported higher satisfaction with physicians' care compared to mothers of term‐born infants. However, delayed cuddling and lower birth weight percentile were associated with lower satisfaction with midwifery care.

Many studies have shown that parents of individuals born preterm often struggle with mental health problems (Pace et al. 2016; Nguyen et al. 2023). A retrospective study of transfers from a New Zealand hospital to a psychiatric consultation team at a children's hospital in New Zealand revealed that 70% of these cases involved mothers of preterm infants (Penny et al. 2015), highlighting the increased need for psychosocial support during their hospital stay. Because access to psychologists in the Neonatal Intensive Care Unit (NICU) is often limited, physicians and nurses play an especially crucial role in providing emotional support. However, this responsibility is further complicated by staff shortages and time constraints (Davidson et al. 2017; Treyvaud et al. 2019).

Social support could be of particular importance to help parents cope with the stressful situation. Griffin and Pickler (2011) highlighted the importance of ongoing support during the transition from hospital to home for mothers of preterm infants, emphasising the need for continuous monitoring and support to help mothers manage the challenges of caring for their infants at home. This aligns with the qualitative findings of a study involving eight fathers and eight mothers in which the parents stated that they could better deal with the situation if there was space in the NICU for them to express their opinions and feelings and that they were heard (Hagen et al. 2016). Mothers of preterm infants with greater access to both formal and informal social support reported lower levels of depressive symptoms. Specifically, the availability of emotional support played a crucial role in mitigating depressive symptoms (Leahy‐Warren et al. 2020).

The findings indicate a desire among mothers of preterm infants for increased medical support, particularly after discharge. At this point, parents of preterm infants often no longer have access to comprehensive medical support despite ongoing health issues. Rehospitalisations often occur, placing additional emotional strain on the parents (Aykanat Girgin and Cimete 2017). In the present study, there was also a desire for more administrative support among mothers of premature babies and those who had experienced delayed cuddling. This aligns with previous research showing that the transition from hospital to home is a critical period where parents require more detailed information, guidance and access to resources to manage their child's ongoing medical needs (Schuetz Haemmerli et al. 2022, Boykova 2016).

Also, mothers who experienced delayed cuddling or whose infants had a low birth weight percentile reported lower satisfaction with midwifery care after hospital discharge, which may be related to unmet expectations around postpartum support. Studies indicate that mothers in the early postpartum period often require personalised and consistent support from midwives, particularly when encountering breastfeeding challenges (Swerts et al. 2019; Ekström and Thorstensson 2015). Effective breastfeeding support from midwives involves not only technical assistance but also emotional encouragement, fostering maternal confidence (Bäckström et al. 2010). However, staff shortages limit their ability to provide extensive one‐on‐one support (Gleeson et al. 2014). When midwives are unable to meet these needs due to limited time, mothers may feel isolated or undersupported. This is particularly relevant for mothers who experienced delayed cuddling or had infants with a low birth weight percentile, as they often feel greater anxiety about their infant's health (Auslander et al. 2003) and may therefore desire more intensive support and reassurance.

The comparison between maternal and paternal responses revealed differences in their support needs, indicating that the experience of preterm birth impacts mothers and fathers differently (Genova et al. 2022). Fathers of preterm infants expressed a desire for administrative support, similar to mothers. In contrast, a greater need for social support was only reported by fathers who experienced delayed cuddling with their child. This suggests that for fathers, the heightened need for social support is linked less to preterm birth itself and more to bonding challenges caused by delayed physical contact. Delayed cuddling may heighten fathers' awareness of emotional needs and bonding difficulties, leading them to seek more social support. Mothers, in contrast, tend to seek social support both in response to premature birth and to the challenges of delayed cuddling.

The subgroup analysis examining the perceived support from the mothers' and children's care networks in recent years revealed a decline in the level of perceived support from the care network. This may be due to several factors, including the increasing staff shortage (Patry et al. 2014), particularly in the medical and nursing fields, which may reduce the time available for providing high‐quality support and care. Additionally, advances in medical technology have resulted in the survival of more extremely premature infants who require particularly intensive levels of support (Glass et al. 2015). Lastly, a potential recall bias could also influence these findings, although efforts were made to mitigate this by limiting the analysis to mothers of children under 18 years of age.

The findings of this study highlight ongoing unmet support needs, particularly in the form of a reported desire for more administrative, social and medical support among mothers of premature infants and those who experienced delayed cuddling. Our study extends the understanding that prematurity increases the vulnerability of infants and caregivers by identifying specific support needs, their variation across gestational age and the association of delayed cuddling with maternal perceptions of care quality. By systematically combining maternal self‐reports on support needs and satisfaction with early perinatal variables, our data offer valuable insights into which subgroups may be at higher risk of unmet needs. Mothers of preterm infants or those who experienced delayed cuddling more often reported a desire for administrative, medical and social support. These results point to concrete opportunities for intervention, particularly in the early postnatal period when responsive and well‐coordinated care is essential. Future support structures should implement tailored, multidisciplinary approaches, especially for mothers experiencing increased stress due to perinatal complications. Early physical contact should also be recognised as an important factor in shaping positive care experiences.

## 5 Strengths and Limitations

This study has several limitations. First, the results may not be generalisable as the research was conducted at a single centre and involved a hospital‐based cohort. Challenges in reaching participants, along with some choosing to opt out, may have introduced selection bias. This study was conducted within the context of the German healthcare system with universal health insurance; therefore, the reported experiences of parents of preterm individuals might not be transferable to other settings such as the US‐American context. The exploratory nature of the study meant that corrections for multiple testing were not applied. Additionally, the use of self‐report questionnaires and retrospective reporting by both mothers and fathers could have led to recall bias.

Nonetheless, this study also has notable strengths as it examined a remarkably large cohort of parents of children born preterm, thus including several generations and stretching across decades, encompassing various degrees of prematurity and including a substantial control group. The multi‐informant design with descriptions provided by both mothers and fathers adds depth to the findings and expands on previous research. A thorough assessment of perinatal medical history was also conducted by reviewing medical charts.

## 6 Conclusion

This study underscores the complex challenges faced by mothers of premature infants and those who experience delayed cuddling, highlighting their increased need for comprehensive support across several domains: administrative, medical and social. These findings call for enhanced postnatal care strategies that are better tailored to the specific needs of these vulnerable families, ensuring that both the medical and emotional needs of parents and children are met during a crucial developmental period.

The results also highlight the disparity between perceived care from different healthcare providers, suggesting that while physicians are often rated highly, midwives may need additional resources to better support mothers of preterm infants, particularly those with delayed cuddling experiences. This points to a need for targeted improvements in midwifery care and staffing. These insights emphasise the critical importance of addressing these gaps to ensure that the diverse needs of these mothers are met with comprehensive and effective support strategies.

## Author Contributions

**Achim Fieß:** conceptualization, formal analysis, validation, writing – original draft, writing – review and editing. **Alica Hartmann:** formal analysis, validation, writing – original draft, writing – review and editing. **Alexander K. Schuster:** conceptualization, formal analysis, validation, writing – review and editing. **Stephanie D. Grabitz:** formal analysis, validation, writing – review and editing. **Dirk Wackernagel:** formal analysis, validation, writing – review and editing. **Michael S. Urschitz:** formal analysis, validation, writing – review and editing. **Jonas Tesarz:** formal analysis, validation, writing – review and editing. **Manfred E. Beutel:** formal analysis, validation, writing – review and editing. **Mareike Ernst:** formal analysis, validation, writing – review and editing. **Eva Mildenberger:** formal analysis, validation, writing – review and editing. **Sandra Gißler:** formal analysis, validation, writing – review and editing.

## Ethics Statement

All participants provided written informed consent before participation, adhering to the standards of Good Clinical Practice, Good Epidemiological Practice and the ethical guidelines of the Declaration of Helsinki. The study protocol and related documents were approved by the local ethics committee of the Medical Chamber of Rhineland‐Palatinate, Germany, under Reference No. 2019‐14161 (original approval: 29.05.2019; most recent update: 02.04.2020) and Reference No. 2021‐15830 (original approval: 05.05.2021; latest update: 19.01.2022).

## Conflicts of Interest

A.K.S. holds the professorship for ophthalmic healthcare research endowed by ‘Stiftung Auge’ and financed by ‘Deutsche Ophthalmologische Gesellschaft’ and ‘Berufsverband der Augenärzte Deutschlands e.V’. A.K.S. receives research support from Allergan, Bayer, Heidelberg Engineering, PlusOptix and Norvartis.

## Supporting information


**Figure S1:** Study design of inclusion and exclusion criteria.
**Figure S2:** Declining trend in mothers' perceived support from the care network by child's birth year. *Note:* The more recently the child was born, the lower the perceived support as reported by the mothers (using the validated German Paediatric Integrated Care Survey [PICS‐D]). To illustrate long‐term trends in maternal perceived support, scores from the ‘Impact on the Family’ and ‘Team Quality & Communication’ subscales were combined into a composite indicator. This representation is exploratory and intended to visualise general developments over time.
**Table S1:** PICS‐D items. The following items were part of the German Paediatric Integrated Care Survey (PICS‐D). Item allocation to the respective PICS‐D scales (e.g., Team Quality & Communication and Family Impact) follows the classification in *Willems J, Bablok I, Sehlbrede M, Farin‐Glattacker E and Langer T (2022) The German paediatric integrated care survey (PICS‐D): Translation, adaptation, and psychometric testing. Front. Pediatr. 10:1057256. doi: 10.3389/fped.2022.1057256*.
**Table S2:** Association analyses of birth experiences of maternal and paternal participants with children born preterm and full‐term. Adjusted for age of mother, father and child.

## Data Availability

Data are available upon reasonable request. Access to data, responsibility and analysis: The analysis presents the clinical data of a cohort. This project constitutes a major scientific effort with high methodological standards and detailed guidelines for analysis and publication to ensure scientific analyses are at the highest level; therefore, data are not made available for the scientific community outside the established and controlled workflows and algorithms. To meet the general idea of verification and reproducibility of scientific findings, we offer access to data at the local database upon request at any time. Interested researchers should make their requests to the coordinating PI of the GPS (Achim Fieβ; achim.fiess@unimedizin-mainz.de). More detailed contact information is available at the homepages of the UM (www.unimedizin‐mainz.de).
