# Supplementary material for: Impact of Premature Birth and Delayed Cuddling on Maternal Support Needs and Satisfaction With Postnatal Care and Changes in Support Over Time
Source: Child Care Health Dev. 2025 Nov 20;51(6):e70170. doi: 10.1111/cch.70170 (PMC12631720; doi:10.1111/cch.70170)
Supplement: Supplementary file 1 — Figure S1: Study design of inclusion and exclusion criteria. Figure S2: Declining trend in mothers' perceived support from the care network by child's birth year. Note: The more recently the child was born, the lower the perceived support as reported by the mothers (using the validated German Paediatric Integrated Care Survey [PICS‐D]). To illustrate long‐term trends in maternal perceived support, scores from the ‘Impact on the Family’ and ‘Team Quality & Communication’ subscales were combined into a composite indicator. This representation is exploratory and intended to visualise general developments over time. Table S1: PICS‐D items. The following items were part of the German Paediatric Integrated Care Survey (PICS‐D). Item allocation to the respective PICS‐D scales (e.g., Team Quality & Communication and Family Impact) follows the classification in Willems J, Bablok I, Sehlbrede M, Farin‐Glattacker E and Langer T (2022) The German paediatric integrated care survey (PICS‐D): Translation, adaptation, and psychometric testing. Front. Pediatr. 10:1057256. doi: 10.3389/fped.2022.1057256. Table S2: Association analyses of birth experiences of maternal and paternal participants with children born preterm and full‐term. Adjusted for age of mother, father and child. [file CCH-51-e70170-s001.docx]

**Figure S1.** Study Design of inclusion and exclusion criteria.

**
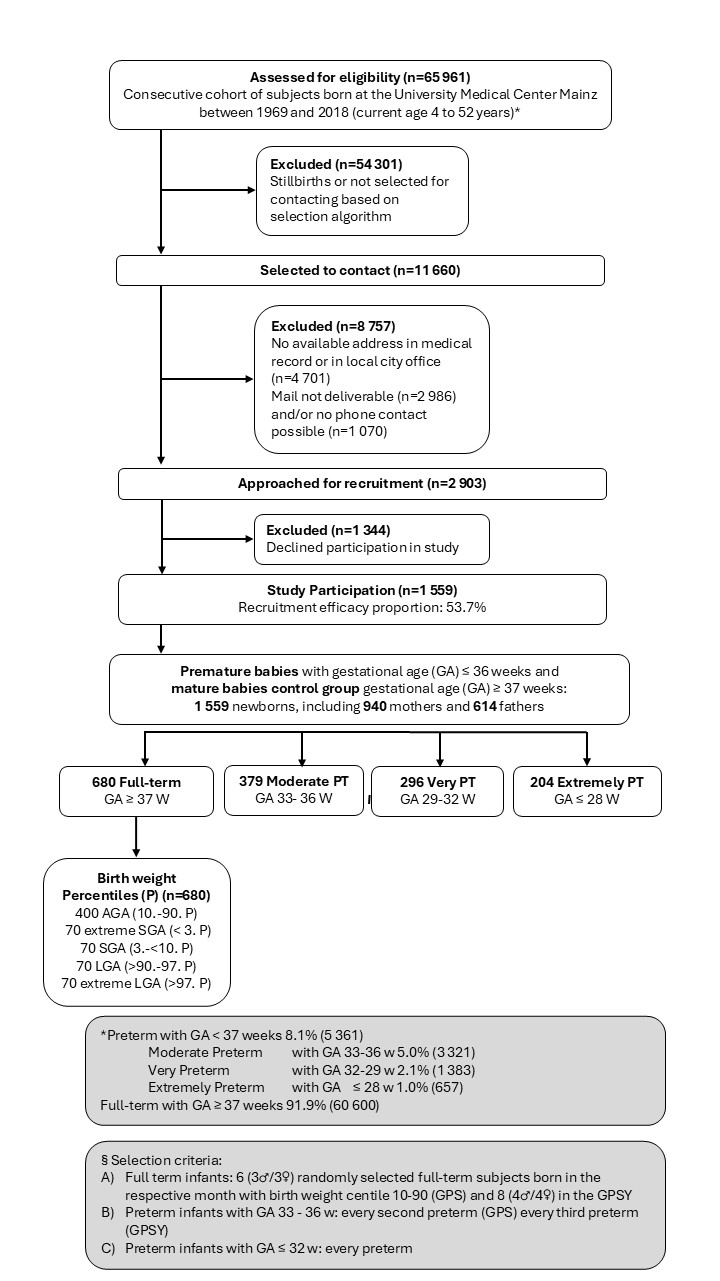
**

**Legend:** PT – preterm; W – weeks

**Figure S2.** Declining trend in mothers' perceived support from the care network by child's birth year.


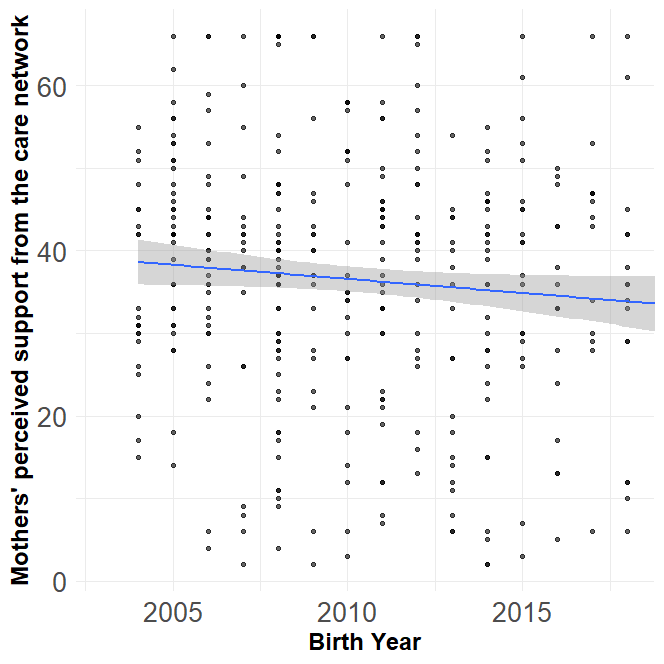


Figure legend: The more recently the child was born, the lower the perceived support as reported by the mothers (using the validated German Pediatric Integrated Care Survey (PICS-D)). To illustrate long-term trends in maternal perceived support, scores from the 'Impact on the Family' and 'Team Quality & Communication' subscales were combined into a composite indicator. This representation is exploratory and intended to visualize general developments over time.

**Table S1. – PICS-D Items**

The following items were part of the German Paediatric Integrated Care Survey (PICS-D). Item allocation to the respective PICS-D scales (e.g., Team Quality & Communication, Family Impact) follows the classification in *Willems J, Bablok I, Sehlbrede M, Farin-Glattacker E and Langer T (2022) The German pediatric integrated care survey (PICS-D): Translation, adaptation, and psychometric testing. Front. Pediatr. 10:1057256. doi: 10.3389/fped.2022.1057256*

| **No. of item in original PISC** | **Original German Question** | **English Translation** |
| --- | --- | --- |
| Item 13 | Hatten alle Mitglieder des Versorgungsnetzes Zugang zu den für sie wichtigen medizinischen Unterlagen (z.B. Arztbriefe)?  Antwortmöglichkeiten: Ja/Nein | Did all members of the care network have access to the medical records that were important to them (e.g. doctor's letters)?  Possible answers: Yes/no |
| Item 22 | Wie oft haben die Mitglieder des Versorgungsnetzes Ihnen Dinge so erklärt, dass Sie sie verstehen konnten? z.B. medizinische Begriffe, Therapiedurchführung, Anwendung von Hilfsmitteln  Antwortmöglichkeiten: Nie/Selten/Manchmal/Meistens/Fast immer/Immer | How often have the members of the care network explained things to you in a way that you could understand? e.g. medical terms, therapy implementation, use of aids  Possible answers: Never/rarely/sometimes/mostly/almost always/always |
| Item 23 | Wie oft hatten Sie das Gefühl, dass Behandlungsempfehlungen zwischen den Mitgliedern des Versorgungsnetzes weitergegeben wurden?  Antwortmöglichkeiten: Nie/Selten/Manchmal/Meistens/Fast immer/Immer | How often did you feel that treatment recommendations were passed between members of the care network?  Possible answers: Never/rarely/sometimes/mostly/almost always/always |
| Item 24 | Wie oft konnten Sie sich gegenüber Mitgliedern des Versorgungsnetzes öffnen oder ihnen mitteilen, dass Sie Sorgen wegen der Gesundheit oder der Betreuung Ihres Kindes hatten?  Antwortmöglichkeiten: Nie/Selten/Manchmal/Meistens/Fast immer/Immer | How often were you able to open up to members of the care network or tell them that you had concerns about your child's health or care?  Possible answers: Never/rarely/sometimes/mostly/almost always/always |
| Item 25 | Wie oft hatten Sie das Gefühl, dass Sie von den Mitgliedern des Versorgungsnetzes gehört wurden, wenn Sie etwas zur Gesundheit Ihres Kindes zu sagen hatten?  Antwortmöglichkeiten: Nie/Selten/Manchmal/Meistens/Fast immer/Immer | How often did you feel that you were heard by members of the care network when you had something to say about your child's health?  Possible answers: Never/rarely/sometimes/mostly/almost always/always |
| Item 26 | Wie oft hat ein Mitglied des Versorgungsnetzwerks Ihnen erklärt, wer für die verschiedenen Bereiche der Versorgung und Betreuung Ihres Kindes verantwortlich ist?  Antwortmöglichkeiten: Nie/Selten/Manchmal/Meistens/Fast immer/Immer | How often has a member of the care network explained to you who is responsible for the different areas of your child's care?  Possible answers: Never/rarely/sometimes/mostly/almost always/always |
| Item 27 | Wie oft hatten Sie das Gefühl, dass die Mitglieder des Versorgungsnetzes über alle bisherigen Tests und Untersuchungen Bescheid wussten, um unnötige weitere Untersuchungen zu vermeiden?  Antwortmöglichkeiten: Nie/Selten/Manchmal/Meistens/Fast immer/Immer | How often did you feel that members of the care network were aware of all previous tests and investigations to avoid unnecessary further investigations?  Possible answers: Never/rarely/sometimes/mostly/almost always/always |
| Item 28 | Wie oft hatten Sie das Gefühl, dass die Mitglieder des Versorgungsnetzes ihre Verantwortung in der Versorgung und Betreuung Ihres Kindes voll und ganz wahrgenommen haben?  Antwortmöglichkeiten: Nie/Selten/Manchmal/Meistens/Fast immer/Immer | How often did you feel that the members of the care network fully assumed their responsibilities in the care and support of your child?  Possible answers: Never/rarely/sometimes/mostly/almost always/always |
| Item 29 | Wie oft hatten Sie das Gefühl, dass die Mitglieder des Versorgungsnetzes Ihr Kind ganzheitlich betrachtet haben, d.h. alle Bedürfnisse Ihres Kindes im Blick hatten?  Antwortmöglichkeiten: Nie/Selten/Manchmal/Meistens/Fast immer/Immer | How often did you feel that the members of the care network took a holistic view of your child, i.e. had all your child's needs in mind?  Possible answers: Never/rarely/sometimes/mostly/almost always/always |
| Item 31 | Haben die Mitglieder des Versorgungsnetzes Ihres Kindes langfristige Behandlungs- oder Betreuungsziele festgelegt, die für 6 Monate oder länger gelten? z.B. Ernährungsplan, Medikamente, Ziele der körperlichen und sprachlichen Entwicklung  Antwortmöglichkeiten: Ja/Nein | Have the members of your child's care network established long-term treatment or care goals that are in place for 6 months or longer? e.g., feeding plan, medications, physical and language development goals  Possible answers: Yes/No |
| Item 32 | Wie oft haben die Mitglieder des Versorgungsnetzes Sie in der Versorgung und Betreuung Ihres Kindes als Partner auf Augenhöhe behandelt?  Antwortmöglichkeiten: Nie/Selten/Manchmal/Meistens/Fast immer/Immer | How often have the members of the care network treated you as an equal partner in the care and support of your child?  Possible answers: Never/rarely/sometimes/mostly/almost always/always |
| Item 33 | Wie oft haben die Mitglieder des Versorgungsnetzes mit Ihnen darüber gesprochen, wie sich Entscheidungen zur Versorgung Ihres Kindes auf Ihre ganze Familie auswirken?  Antwortmöglichkeiten: Nie/Selten/Manchmal/Meistens/Fast immer/Immer | How often have members of the care network spoken to you about how decisions about the care of your child affect your whole family?  Possible answers: Never/rarely/sometimes/mostly/almost always/always |
| Item 34 | Wie oft haben die Mitglieder des Versorgungsnetzes mit Ihnen über Belastungen gesprochen, die sich für Sie durch die Erkrankung und den betreuungsbedarf Ihres Kindes ergeben?  Antwortmöglichkeiten: Nie/Selten/Manchmal/Meistens/Fast immer/Immer | How often have the members of the care network spoken to you about the burdens that arise for you as a result of your child's illness and care needs?  Possible answers: Never/rarely/sometimes/mostly/almost always/always |
| Item 35 | Wie oft haben die Mitglieder des Versorgungsnetzes Situationen angesprochen, die es ihnen schwer machen sich um die Gesundheit Ihres Kindes zu kümmern? z.B. Arbeit, finanzielle Sorgen, eigene Krankheit  Antwortmöglichkeiten: Nie/Selten/Manchmal/Meistens/Fast immer/Immer | How often have members of the care network addressed situations that make it difficult for them to look after their child's health? e.g. work, financial worries, own illness  Possible answers: Never/rarely/sometimes/mostly/almost always/always |
| Item 36 | Wie oft haben die Mitglieder des Versorgungsnetzes angeboten, auch auf andere Weise als durch einen persönlichen Besuch mit Ihnen zu kommunizieren, wenn keine körperliche Untersuchung Ihres Kindes notwendig war? z.B. Telefon, E-mail, Skype  Antwortmöglichkeiten: Nie/Selten/Manchmal/Meistens/Fast immer/Immer | How often have members of the care network offered to communicate with you in ways other than an in-person visit when a physical examination of your child was not necessary? e.g. phone, email, Skype  Possible answers: Never/rarely/sometimes/mostly/almost always/always |
| Item 37 | Wie oft haben die Mitglieder des Versorgungsnetzes Ihnen Möglichkeiten geboten, mit anderen betroffenen Familien in Kontakt zu treten?  Antwortmöglichkeiten: Nie/Selten/Manchmal/Meistens/Fast immer/Immer | How often have the members of the care network offered you opportunities to get in touch with other affected families?  Possible answers: Never/rarely/sometimes/mostly/almost always/always |

**Table S2**. Association analyses of birth experiences of maternal and paternal participants with children born preterm and full-term. Adjusted for age of mother, father and child.

|  | **Multivariable model**  **adjusted for age of parents and child** | | | |
| --- | --- | --- | --- | --- |
|  | Mother | | Father | |
|  | OR (CI_95_) | p | OR (CI_95_) | p |
| **More**  **administrative support** | | | | |
| Weeks of prematurity  (40 minus GA) | 1.10  (1.02, 1.20) | 0.02 | 1.12  (1.00, 1.26) | 0.06 |
| BW percentile | 0.99  (0.98, 1.01) | 0.28 | 1.01  (0.99, 1.03) | 0.30 |
| Cuddling after birth (weeks) | 1.09  (0.83, 1.40) | 0.49 | 0.90  (0.48, 1.34) | 0.68 |
| **More social support** | | | | |
| Weeks of prematurity  (40 minus GA) | 1.07  (1.01, 1.13) | 0.02 | 1.03  (0.95, 1.13) | 0.45 |
| BW percentile | 1.00  (0.99, 1.00) | 0.39 | 1.00  (0.99, 1.02) | 0.62 |
| Cuddling after birth (weeks) | 1.31  (1.08, 1.63) | 0.01 | 1.29  (1.00, 1.65) | 0.04 |
| **More medical support** | | | | |
| Weeks of prematurity  (40 minus GA) | 1.01  (0.95, 1.07) | 0.77 | 1.04  (0.97, 1.12) | 0.25 |
| BW percentile | 1.00  (1.00, 1.01) | 0.26 | 1.00  (0.99, 1.01) | 0.69 |
| Cuddling after birth (weeks) | 1.37  (1.12, 1.71) | 0.003 | 1.06  (0.80, 1.34) | 0.64 |
| **More economic support** | | | | |
| Weeks of prematurity  (40 minus GA) | 0.99  (0.91, 1.06) | 0.72 | 1.01  (0.94, 1.09) | 0.69 |
| BW percentile | 1.01  (1.00, 1.02) | 0.30 | 1.01  (1.00, 1.02) | 0.04 |
| Cuddling after birth (weeks) | 1.33  (1.07, 1.66) | 0.01 | 1.10  (0.83, 1.39) | 0.43 |
| Legend: ICU stay – Intensive care unit stay; BW – birth weight; GA – gestational age | | | | |
